# The feasibility of a “no tube, no fasting” fast-track recovery protocol after esophagectomy for esophageal cancer patients aged 75 and over

**DOI:** 10.3389/fonc.2023.1144047

**Published:** 2023-05-18

**Authors:** Wenqun Xing, Xianben Liu, Peng Miao, Wentao Hao, Keting Li, Hao Wang, Yan Zheng

**Affiliations:** ^1^ Department of Thoracic Surgery, The Affiliated Cancer Hospital of Zhengzhou University, Henan Cancer Hospital, Zhengzhou, China; ^2^ Department of Thoracic Surgery, Henan Provincial People’s Hospital, People’s Hospital of Zhengzhou University, Zhengzhou, China; ^3^ Department of Thoracic Surgery, Zhongshan Hospital of Fudan University, Shanghai, China

**Keywords:** elderly patients, no tube, no fasting, minimally invasive esophagectomy, early oral feeding

## Abstract

**Objective:**

For elderly patients aged ≥75 with esophageal cancer, whether surgical treatment is safe and effective and whether it is feasible to use a relatively radical “no tube, no fasting” fast-track recovery protocol remain topics of debate. We conducted a retrospective analysis to shed light on these two questions.

**Methods:**

We retrospectively collected the data of patients who underwent McKeown minimally invasive esophagectomy (MIE) combined with early oral feeding (EOF) on postoperative day 1 between April 2015 and December 2017 at Medical Group 1, Ward 1, Department of Thoracic Surgery of our hospital. Preoperative characteristics, postoperative complications, operation time, intraoperative blood loss, duration of anastomotic leakage (day), hospital stay, and survival were evaluated.

**Results:**

Twenty-three elderly patients with esophageal cancer underwent surgery with EOF. No significant difference was observed in intraoperative measures. The incidence of postoperative complications was 34.8% (8/23). Two patients (8.7%) were terminated early during the analysis of the feasibility of EOF. For all 23 patients, the mean hospital stay was 11.4 (5-42) days, and the median survival was 51 months.

**Conclusion:**

Patients aged ≥75 with resectable esophageal cancer can achieve long-term survival with active surgical treatment. Moreover, the “no tube, no fasting” fast-track recovery protocol is safe and feasible for elderly patients.

## 1 Introduction

Esophageal cancer is the ninth most common malignant tumor and the sixth leading cause of death in the world (1, 2). At present, surgery is the main treatment for esophageal cancer. However, for elderly (≥75 years) patients with esophageal cancer, conservative treatments such as radiotherapy and chemotherapy are usually preferred because of advanced age (which may increase the risk of death) and other considerations (high incidence of postoperative complications, long recovery time, poor prognosis). Nevertheless, a retrospective study in Belgium demonstrated that carefully selected elderly patients with esophageal cancer can achieve good prognosis after traditional open surgery (3). Compared with traditional open surgery, minimally invasive esophagectomy (MIE) has the advantages of less trauma and fewer complications (4) and has gradually become the main surgical method for esophagectomy. Our previous randomized controlled trial (RCT) showed that it is feasible to perform McKeown MIE combined with the “no tube, no fasting” fast-track recovery protocol and that early oral feeding (EOF) after operation does not increase complications and can minimize the pain and discomfort associated with tubing and reduce the hospital stay (5), thereby facilitating fast recovery. Is MIE combined with the “no tube, no fasting” fast-track recovery protocol feasible for elderly patients?

In this study, we retrospectively analyzed the short-term complications and long-term outcomes of elderly patients with esophageal cancer who underwent MIE combined with the “no tube, no fasting” fast-track recovery protocol at our department between 2015 and 2018 to investigate the feasibility of the “no tube, no fasting” fast-track recovery protocol for elderly patients.

## 2 Patients and methods

**Figure 1:**
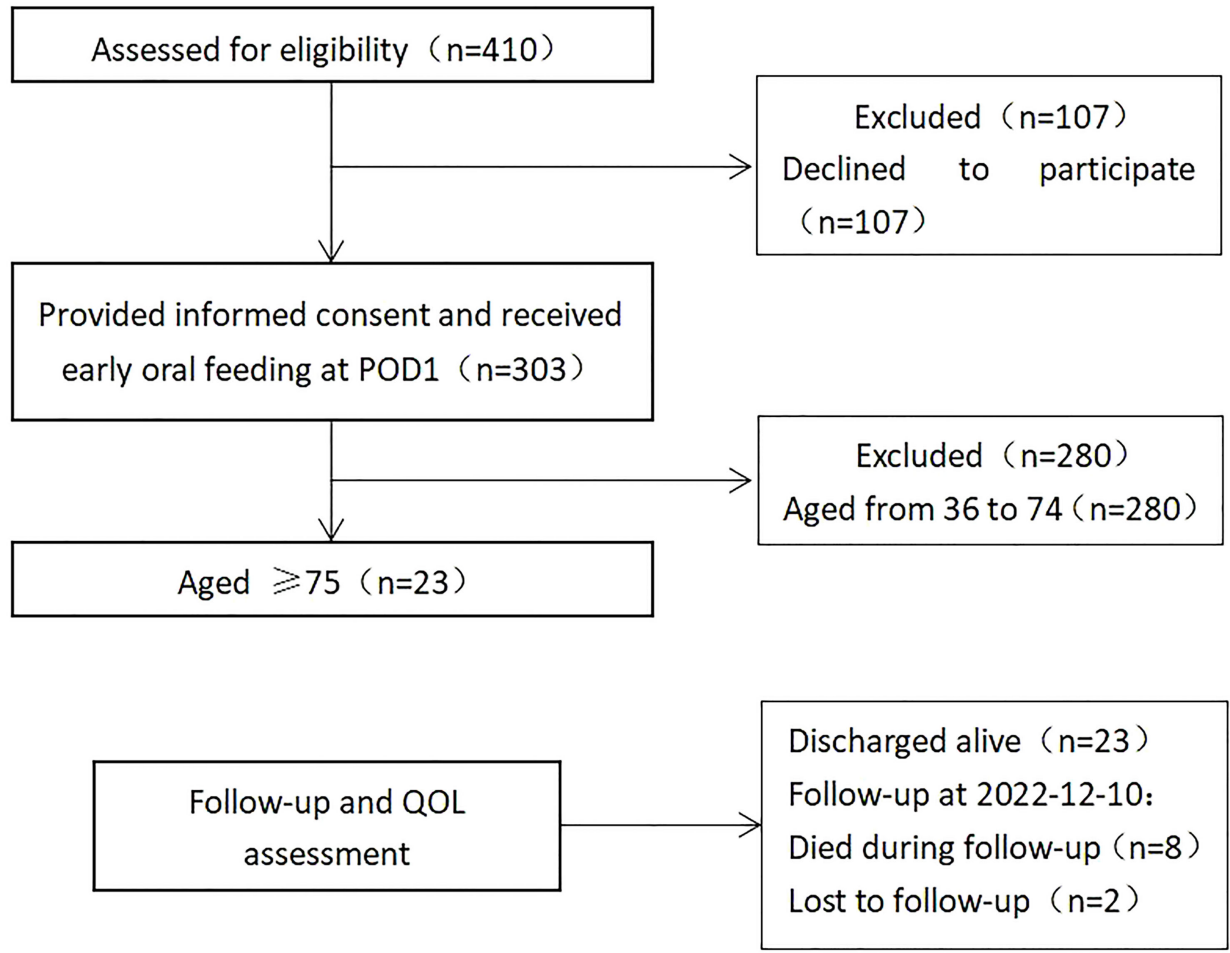
Screening process for enrolled patients.

We retrospectively analyzed the data of 410 patients with resectable thoracic esophageal cancer treated at Medical Group 1, Ward 1, Department of Thoracic Surgery, Cancer Hospital Affiliated to Zhengzhou University/Henan Cancer Hospital between April 2015 and December 2017. The number of patients who ate food on the first postoperative day was 303, and patients younger than 75 years old were excluded, so 23 patients were finally enrolled (**Figure 1**). This study was formally approved by the Ethics Committee of our hospital. Participation was voluntary, and each patient signed written informed consent before the study. The inclusion criteria were as follows: (i) aged ≥75; (ii) histologically-confirmed esophageal squamous cell carcinoma or esophageal adenocarcinoma; (iii) adequate organ function; and (iv) patients who followed the “no tube, no fasting” fast-track recovery protocol after operation. The exclusion criteria were as follows: (i) patients contraindicated for McKeown MIE due to tumor progression; (ii) severe underlying diseases such as significant liver cirrhosis, diabetes with organ damage, or severe cardiovascular or renal diseases; or (iii) severe malnutrition [body mass index <15 kg/m² (6)]. The patient underwent routine preoperative chest and abdominal enhanced computed tomography (CT), endoscopic ultrasound, head CT neck color ultrasound, heart color ultrasound, upper digestive tract angiography and other examinations. Positron emission tomography (PET) examination was used when distant organ metastasis was suspected; lymph node puncture biopsy was performed when cervical lymph node metastasis was suspected.All cases were staged according to the 7th Edition of the Classification of Tumor, Lymph Node Metastasis, and Metastasis of Malignant Tumors (7). Postoperative complications were graded with the Clavien-Dindo grading system. A multidisciplinary team performed a comprehensive evaluation of each patient’s condition before surgery to develop the best treatment plan.

### 2.1 Ethics statement

Studies involving animal subjects

Generated Statement: No animal studies are presented in this manuscript.

Studies involving human subjects

Generated Statement: The studies involving human participants were reviewed and approved by Ethics Review Committee of the Affiliated Cancer Hospital of ZhengZhou University/Henan Cancer Hospital. The patients/participants provided their written informed consent to participate in this study.

Inclusion of identifiable human data

Generated Statement: No potentially identifiable human images or data is presented in this study.

## 3 Surgery

All patients underwent McKeown MIE combined with the “no tube, no fasting” fast-track recovery protocol. First, the patient was placed in the left decubitus position with 30° anteversion. The thoracic esophagus was carefully dissected, and lymph nodes collected. Next, the patient was placed in a supine position with the head turned to the right. A 2- to 3-cm incision was made in the left neck to expose and incise the cervical esophagus. Next, five ports were placed in the abdominal wall, and the stomach was carefully mobilized. Care was taken to preserve the right gastroepiploic pedicle. Once fully mobilized, the stomach was pulled out through a small incision (median, 3-4 cm) in the abdominal wall, and linear cutting staplers (TLC, Ethicon, Cincinnati, Ohio, USA) were used to prepare a 4 cm gastric tube. Gastric emptying was not performed during operation. Next, the gastric tube was lifted up to the neck and sutured with the distal esophagus (see Li et al. for anastomosis technique) (8, 9). No nasogastric tube was placed before or after operation. All patients started EOF with their preferred foods on postoperative day 1.On day 1, the patients were allowed to have a liquid or semi-liquid diet, they should take a cup of warm water and be observed whether the patients coughed or choked. If not, a nutritionist would guide them to drink milk, juice and gruel. If coughing occured after drinking water, changed to semi-liquid food with high viscosity, such as soft noodles, rice porridge, etc., continued to observe the eating reaction, if coughing still occured, they need to stopp eating. On the second day, semi-liquid diet and soft solid diet such as soft cake, soft noodles, rice, eggs and soft steamed buns were allowed. The patients were encouraged to chew carefully and thoroughly. During feeding, the patient was monitored by at least one clinician or nutritionist. Feeding was discontinued immediately in case of any aspiration symptoms such as persistent coughing. If this happened during postoperative days 1-3, a nutritionist calculated the patient’s calorie intake and ordered intravenous supplementation if warranted. At the same time, indwelling naso-jejunal feeding tube was required.Intravenous infusion was discontinued on postoperative day 4. Gastrointestinal decompression was performed in case of an anastomotic leak or severe distension.In this study, standardized clinical approaches were adopted for postoperative management of all patients. The discharge criteria were as follows: normal vital signs, no signs of postoperative complications requiring hospitalization, ambulatory without assistance, and pain that could be tolerated with oral analgesia (5).

The Windows version of SPSS 25.0 (SPSS Inc, Chicago, IL, USA) was used for statistical analysis. The patient’s survival status at the last follow-up was defined as the survival outcome. Survival was defined as the time from initial McKeown MIE to death or December 10, 2021. The Kaplan-Meier method was used to plot survival curves.

## 4 Results

### 4.1 Short-term Complications

Among the 23 patients, 21 patients (91%) had esophageal squamous cell carcinoma, and two patients (9%) had esophageal adenocarcinoma; four patients (17%) received preoperative neoadjuvant therapy, and one patient received preoperative and postoperative adjuvant chemotherapy (**Table 1**); 15 patients had comorbidities before operation, eight of whom had varying severities of hypertension. The operation was successful in all 23 patients. The median patient age was 78 (75–84) years, the mean operation time was 217 (180-365; median: 205) minutes, and the mean blood loss was 65 (30-200; median: 50) mL. The mean time to initial postoperative gas-passing/bowel movement was 2.9 (1-5; median: 3) days. Eight patients (35%) underwent thoracic duct ligation. The median number of lymph nodes dissected was 31 (0-52). The mean time to remove the mediastinal tube was 8.3 (5-30; median: 7) days after operation (**Table 2**). These results are consistent with our previous findings from 140 EOF patients (5). The mean hospital stay was 11.4 (5-42 days; median: 9) days in elderly patients, which was longer than the mean hospital stay of younger patients (7 [7-8] days) (n ¼ 140).

**Table 1:** Baseline demographic and clinical characteristics of esophageal carcinoma patients.

Characteristic	EOF Group (n=23)
**Mean Age (range)**	78 (75-86)
Sex	
** Male**	12 (52%)
** Female**	11 (48%)
Location of tumor	
** Upper**	6 (26%)
** Middle**	8 (35%)
** Lower**	9 (39%)
Histology	
** Squamous cell carcinoma**	21 (91%)
** Adenocarcinoma**	2 (9%)
Differentiation	
** High**	4 (17%)
** Moderate**	9 (39%)
** Poor**	10 (44%)
cTNM	
** 0-II**	10 (43%)
** III**	13 (57%)
pT/ypT	
** 0**	2 (9%)
** 1**	6 (26%)
** 2**	3 (13%)
** 3**	12 (52%)
pN/ypN	
** 0**	16 (69%)
** 1**	5 (22%)
** 2**	2 (9%)
**Neoadjuvant treatment**	4 (17%)
**adjuvant therapy**	1 (4%)

EOF, early oral feeding; cTNM, clinical tumor/node/metastasis stage; pN, pathological lymph nodes; pTNM, tumor/node/metastasis.

**Table 2:** Intraoperative and postoperative outcome.

Characteristic	EOF Group (n=23)
**Mean operative time (range) (min)**	217 (180-365)
**Mean blood loss (range) (mL)**	65 (30-200)
**Thoracic duct ligation**	8 (35%)
**Median lymph nodes retrieved (range) N**	31 (0-52)
**Median Positive lymph nodes (range) N**	0 (0-3)
**Median Chest tube drainage days (range)**	7 (5-30)
**Mean Length of postoperative stay (d)**	11.4 (5–42)
**Mean Time to first flatus/bowel movement (d)**	2.9 (1-5)

N, number; d, days.

Postoperative complications are summarized in **Table 3**: eight patients (8/23) had cardiac, respiratory, nervous system, gastrointestinal, or anastomotic complications after operation. Respiratory complications were more common, especially pneumonia (n=3), and one patient with pneumonia also had severe respiratory failure. In addition, two patients had recurrent laryngeal nerve damage, and three patients had arrhythmia. Only one patient (4.3%) with severe pneumonia and delayed gastric emptying required therapeutic nasogastric decompression. During the subsequent follow-up, no anastomotic leaks were reported, whereas eight EOF patients (5.7%) required decompression in a previous study (5). Furthermore, two patients (2/23) were readmitted to the ICU. According to the Clavien-Dindo grading system, only these two patients had grade IV postoperative complications (recurrent laryngeal nerve damage [n=1], respiratory failure [n=1]). After EOF on postoperative day 1, two patients with respiratory failure and anastomotic leakage were terminated early. A feeding tube was placed and then removed once patient condition was stabilized. The remaining patients were able to follow the EOF schedule.

**Table 3:** The postoerative complications.

Variable	EOF Group (n =23)
**Complication**	8
**Pneumonia**	3
**Pneumothorax**	2
**Pleural effusions**	3
**Respiratory failure**	1
**Arrhythmia**	3
**Anastomotic leak**	1
**Injury of recurrent nerve**	2
**Delayed gastric emptying**	1
**Clavien-Dindo grading system**	
**I**	3
**II**	2
**III**	1
**IV**	2
**Readmission to ICU**	2

EOF, early oral feeding; N, number.

### 4.2 Long-term outcomes

**Figure 2:**
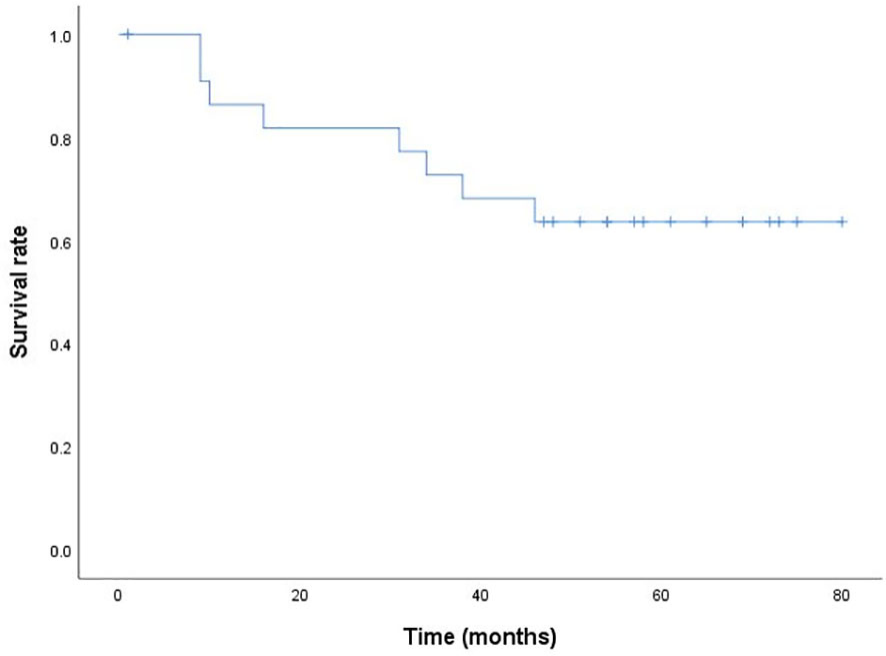
The postoperative cumulative overall five-year survival curve (Kaplan-Meier method).

As of December 10, 2022, 13 patients were alive (two patients were lost to follow-up), five patients died of tumor progression, and three patients died of non-cancer-related causes such as underlying diseases or comorbidities. The survival curves are shown in **Figure 2**. The median survival was 51 (1-80) months.

## 5 Discussion

Due to high perioperative mortality in association with relatively poor nutritional status, complex preoperative comorbidities, and a high incidence of postoperative complications, advanced age is considered a relative contraindication for major surgeries such as esophagectomy (10). Each patient must decide whether to undergo this risky surgery considering their relatively short life expectancy. Researchers are also increasingly investigating the necessity of surgery. A US study retrospectively analyzed the postoperative data of patients with esophageal cancer who underwent surgery in 2000-2014 and found that the annual esophagectomy rate remained largely unchanged at 1544 (29.5%) patients aged ≥70 during the study period (28.4% in 2000, 26.3% in 2014, P=0.76); however, the probability of postoperative heart failure and in-hospital mortality was significantly higher, and the estimated mortality rate was positively correlated with age (1.5% for patients in their 40s, 2.5% in their 50s, 3.6% in their 60s, 5.4% in their 70s, 7.0% in their 80s) (11). In 2013, Markar SR et al. reached similar conclusions that perioperative mortality was greater in elderly patients with esophageal cancer after esophagectomy and that in-hospital mortality was significantly correlated with advanced age. Compared with younger patients, the postoperative 5-year survival rate was lower in the elderly group (29.01% vs 21.23%; pooled odds ratio=0.73; 95% CI=0.62-0.87; P<0.05) (12). Both studies show that the surgical risk is high for elderly patients. Rahouma M et al. retrieved and analyzed relevant data from the US Cancer Registry on patients with locally advanced esophageal cancer who were advised to undergo surgery but refused surgery in 2004-2014. Many patients were elderly. After propensity matching (n=525 in each group), the median survival time was significantly longer in patients who underwent surgery than in patients who refused surgery (32 vs 21 months, P<0.001) (13), indicating that surgery is necessary. Japanese researchers reached similar conclusions that when surgically indicated, esophagectomy is a viable treatment option with satisfactory outcomes even in patients aged 70 years and older and does not increase morbidity or mortality (14). These results are consistent with the findings of this study.

Despite some discordant results, these studies all agree that postoperative complications are important life-threatening factors for elderly patients with resectable esophageal cancer. However, this study demonstrates that among the 23 elderly patients who underwent McKeown MIE combined with the “no tube, no fasting” fast-track recovery protocol, only eight patients (34.8%) experienced related complications, and only one patient had anastomotic leakage, the most common complication after esophagectomy. Studies have shown that the five-year survival rate of all-stage esophageal cancer is approximately 15% (15). However, in this study, the five-year survival rate of elderly patients with esophageal cancer was higher, with a median survival time of 51 months, and the expected life expectancy was significantly extended. Therefore, we believe that McKeown MIE combined with the “no tube, no fasting” fast-track recovery protocol increases the chance of positive outcomes of surgical resection for elderly patients with esophageal cancer and that the weight of age should be reduced accordingly. These data indicate that age may not be an important factor for surgery. It is more important to comprehensively evaluate the patient’s general condition and extent of disease than advanced age (16, 17). Surgical treatment is useful for elderly patients with esophageal cancer.

Moreover, this study demonstrates that the “no tube, no fasting” fast-track recovery protocol is safe and feasible for elderly patients. In general, enteral nutrition (via a nasojejunal or jejunostomy tube) is administered to meet nutrition needs after esophagectomy (18, 19). However, the procedure is complex and causes discomfort. Our previous study showed that patients could start feeding on postoperative day 1 after MIE (20), which also reduced discomfort. For patients aged ≥75, we found that the incidence of anastomotic leakage, the most significant risk of EOF, was extremely low (1/23); patient gastrointestinal function recovered early; and the incidence of postoperative complications was acceptable (8/23), although hospital stay was slightly prolonged.

This study has some limitations. This is a single-center study. The sample was small and may not represent all elderly patients with esophageal cancer. Moreover, the elderly patients included in this study had overall fewer underlying diseases, less severe comorbidities, and a low percentage of neoadjuvant therapy. Therefore, RCTs are needed to investigate the feasibility of our protocol for elderly patients with severe conditions. Furthermore, our surgeons are very experienced; our hospital is located in an area with a high prevalence of esophageal squamous cell carcinoma, and we perform approximately 1000 operations for esophageal cancer patients each year. Further research is needed to investigate whether our conclusions are applicable to low-prevalence areas.

## 6 Conclusion

This study demonstrates that for elderly patients aged ≥75 with resectable esophageal cancer, McKeown MIE (after comprehensive assessment of patient condition) is necessary and enables satisfactory long-term outcomes. Moreover, this study demonstrates the safety and effectiveness of the relatively radical “no tube, no fasting” fast-track recovery protocol with EOF on postoperative day 1. For elderly patients, EOF has been demonstrated to accelerate recovery, effectively alleviate pain, and reduce economic burden. Due to some limitations as the small sample size, further research is needed to validate our results before widespread clinical application.

## Data availability statement

The original contributions presented in the study are included in the article/supplementary material. Further inquiries can be directed to the corresponding author.

## Ethics statement

The studies involving human participants were reviewed and approved by The Affiliated Cancer Hospital of Zhengzhou University/Henan Cancer Hospital. The patients/participants provided their written informed consent to participate in this study.

## Author contributions

(I) Conception and design: WX, YZ. (II) Administrative support: WX, YZ. (III) Provision of study materials or patients: WX, XL, YZ. (IV) Collection and assembly of data: PM, WH (V) Data analysis and interpretation: PM, WH, HW, YZ. (VI) Manuscript writing: All authors (VII) Final approval of manuscript: All authors. 
